# Bioactive Compounds of Dietary Origin and Their Influence on Colorectal Cancer as Chemoprevention

**DOI:** 10.3390/life13101977

**Published:** 2023-09-28

**Authors:** Paulina Delgado-Gonzalez, Elsa N. Garza-Treviño, David A. de la Garza Kalife, Adriana Quiroz Reyes, Esther Alhelí Hernández-Tobías

**Affiliations:** 1 Departamento de Bioquímica y Medicina Molecular, Facultad de Medicina, Universidad Autónoma de Nuevo León (UANL), Monterrey 6440, Mexico; elsa.garzatr@uanl.edu.mx (E.N.G.-T.); david.delagarzaka@gmail.com (D.A.d.l.G.K.); guadalupe.quirozrys@uanl.edu.mx (A.Q.R.); 2 Facultad de Salud Pública y Nutrición, Universidad Autónoma de Nuevo León (UANL), Monterrey 6440, Mexico; esther.hernandeztb@uanl.edu.mx

**Keywords:** colorectal cancer, chemopreventive compounds, molecular pathways

## Abstract

Colorectal cancer (CRC) is one of the most common causes of death and the third most diagnosed cancer worldwide. The tumor microenvironment and cancer stem cells participate in colorectal tumor progression and can dictate malignancy. Nutrition status affects treatment response and the progression or recurrence of the tumor. This review summarizes the main bioactive compounds against the molecular pathways related to colorectal carcinogenesis. Moreover, we focus on the compounds with chemopreventive properties, mainly polyphenols and carotenoids, which are highly studied dietary bioactive compounds present in major types of food, like vegetables, fruits, and seeds. Their proprieties are antioxidant and gut microbiota modulation, important in the intestine because they decrease reactive oxygen species and inflammation, both principal causes of cancer. These compounds can promote apoptosis and inhibit cell growth, proliferation, and migration. Combined with oncologic treatment, a sensitization to first-line colorectal chemotherapy schemes, such as FOLFOX and FOLFIRI, is observed, making them an attractive and natural support in the oncologic treatment of CRC.

## 1. Introduction

Colorectal cancer (CRC) is third in incidence worldwide and second in mortality. One of the main risk factors for developing CRC is a lifestyle with poor physical activity and a diet high in fat and carbohydrates, which stimulates an inflammatory state that increases reactive oxygen species (ROS) and releases proinflammatory cytokines in the intestinal mucosa [1]. Nutritional status influences part of the response to treatment in cancer patients. For example, malnutrition predicts worse results in response to treatment and survival [2]; also, recurrence, progression, and the presence of metastasis have been attributed to poor nutritional status [3].

The bioactive compounds of food have been of interest since they may contribute to maintaining human health. These compounds are present in small amounts in plants and certain foods such as fruits, vegetables, nuts, and seeds. They can interact with tissue components, potentially promoting multiple health benefits [4]. Bioactive compounds have a wide spectrum of chemical structures, and their main function is as antioxidants; they also modulate the expression and activity of transcription growth factors and inflammatory mediators and are cell cycle intermediaries [1,5]. They can modulate immunoregulator molecules such as PD-L1 by modulating mitochondria activity and hypoxia, ubiquitination, glycosylation, and endoplasmic reticulum degradation, among others [6,7,8,9]. It has been proposed to consider the bioavailability of the compound rather than the amount present in food or dietary supplements to determine the benefit of the compound. It has been reported that the DNA damage repair mechanism can activate the expression of PD-L1 since BRCA2 or Ku70/80 depletion enhances PD-L1 upregulation after DSBs in an ATM/ATR/Chk1-dependent manner [10,11,12]. The consumption of various bioactive compounds, such as phenolic compounds, flavonoids, phenolic acids, carotenoids, and xanthophylls, influences the preservation of cell integrity, the maintenance of cell DNA repair systems, and their action in cell differentiation, proliferation, and apoptosis. This review summarizes the origin of bioactive compounds found in dietary sources such as curcumin, quercetin, resveratrol, lycopene, and cinnamic acid. It also explores their remarkable attributes in regulating the metabolic pathways linked to CRC and their significant roles in chemoprevention and reverse chemoresistance.

## 2. Generalities of Bioactive Compounds in CRC

Bioactive products have always played a significant role as novel therapeutical agents, irrespective of their source of origin. They are phenolic compounds, flavonoids, phenolic acids, carotenoids, and xanthophylls. Different concentrations are found in vegetables, fruits, grains, spices, and their derived foods and beverages, such as tea, olive oil, fruit juices, wine, chocolate, and beer, as shown in Table 1.

### 2.1. Polyphenols

Polyphenols are compounds in plants with one or more hydroxyl groups attached to an aromatic ring in their structure [13]. Their bioavailability and stability are low, and most of their metabolism occurs in the large intestine, where bacteria convert them to phenolic acid that cells absorb and use. Polyphenols are divided into flavonoids, quercetin, and epigallocatechin-3-gallate (EGCG). Phenolic acids are derivatives of hydroxybenzoic acid or hydroxycinnamic acid, tannins, lignans, and stilbenes.

Their main properties are antioxidant by suppressing the generation of reactive oxygen species and lipid peroxidation. However, they also have anti-inflammatory, immunomodulatory, anticancer, and gastroprotective effects [14].

One of its proposed mechanisms of action is inhibiting the NF-ĸB transcription factor, which is known to participate in the development of colorectal cancer and intestinal inflammatory diseases and is responsible for regulating inflammation, cell growth, and cell survival [15]. Another mechanism proposed for polyphenols as a bioactive compound against cancer is through the inhibition of proinflammatory enzymes, such as cyclooxygenase 2 (COX2) and 5-lipoxygenase [16], whose altered metabolism is common in neoplasms derived from the epithelium. Polymorphisms in COX1 and COX2 have been associated with the development of colorectal cancer, specifically COX2, which controls the cell cycle [17] and its expression increases in response to cytokines and growth factors. COX2 participates in cancer development by increasing cell proliferation, tumor angiogenesis [18], and the production of prostaglandins that seem to increase resistance to apoptosis in epithelial cells. In contrast, in stromal cells, it favors neovascularization present in the development of CRC.

### 2.2. Flavonoids

Flavonoids are the most abundant polyphenols and participate as proposals, along with conventional treatments, to eradicate different types of cancer, including colorectal cancer, by modulating various enzymes, such as phosphatidylinositol 3 kinase (PI3K), of which class I is involved in cancer [19]. They also modulate AKT/protein kinase B, involved in cell proliferation, survival, and growth, through its effect on the TSC1/TSC2, mTORC, MAPK, and glucose metabolism [20] pathways that participate in the amplification of molecules favoring cell proliferation, growth, and survival and may explain resistance to first-line treatments in CRC, such as irinotecan, 5-fluorouracil (5-FU), and cisplatin [21]. Resveratrol inhibits the paclitaxel-induced neuropathic pain caused by the PI3K/AKT and SIRT1/PGC1α pathways [22]. Urolithin A, a metabolite produced from the dietary polyphenol ellagic acid, potentiates the effects of 5-FU on colon cancer cells by regulating cyclins A and B1 and activating caspases 8 and 9 to trigger cell cycle arrest and apoptosis [23].

A few randomized controlled trials have evaluated the effect of polyphenols on CRC. Sinicrope et al. (2021) carried out a phase II study where they measured the effectiveness of the Poly E bioactive compound of green tea that contains between 55% and 72% of the active component epigallocatechin on the percentage change in the number of rectal aberrant crypts, which are considered premalignant lesions [24]. With a 6-month intervention with 780 mg oral supplementation of Poly E, they found that it did not significantly reduce the number of crypts, but adenoma recurrence decreased by 29% [24]. The antitumor effect on CRC cells in vitro and in vivo are related in a dose-dependent manner [25]. A report demonstrated that a higher intake of total polyphenols, total flavonoids, total phenolic acids, anthocyanin, and flavanols was associated with a reduction in CRC risk and that stilbenes were inversely associated with colorectal adenomas [26].

### 2.3. Carotenoids

Carotenoids, a vitamin A (retinol) source, contain a central carbon chain with alternating single and double bonds and present cyclic or acyclic terminal groups [27]. They are found in fruits, vegetables, and certain fish and are represented as B-carotene, lycopene, lutein, zeaxanthin, and curcuma. They function as antioxidants.

Carotenoids have been used as anti-tumor agents for inhibiting tumor growth, inducing apoptosis, modulating gene expression and the immune response, modulating the AP-1 transcriptional complex, WNT/pathway B catenin, and inhibiting mutagenesis [28], although cancer related to inflammation can, understandably, be hindered by carotenoids with anti-inflammatory effects, describing specific signaling pathways. CRC stem cell treatment with β-carotene reduces cell proliferation by regulating miRNA expression, elevating histone H3 and H4 acetylation, downregulating DNMT3A mRNA, and global DNA methylation [29].

Studies have analyzed the intake impact of dietary carotenoids and CRC [30], finding that high B-carotene intake in men is negatively associated with CRC; hence, it has been proposed as a preventive compound since it can modulate M2 macrophages and fibroblasts, in addition to reducing COX2 [31]. The consumption of lycopene and lutein/zeaxanthin has a non-significant inverse association with CRC. Lycopene, in studies with CRC cell lines [32] inhibits AKT protein phosphorylation and, in the presence of eicosapentaenoic acid, suppresses the P1K3/AKT pathway, blocking mTOR activity [33]. It also improves antitumoral parameters such as catalase, glutathione, and IFN-γ expression [34]. Lycopene reduces the oxidative stress caused by chemotherapeutic 5-FU and oxaliplatin, decreasing the adverse effects of CRC treatment [35]. 

Similarly, astaxanthin, another carotenoid, can upregulate the expression of the epithelial marker E-cadherin and downregulate the mesenchymal markers vimentin and cortactin. It also increases miR-29a-3p and miR-200a expression, suppressing matrix metalloproteinase-2 (MMP2) and thus inhibiting the metastatic potential of CRC [36]. Moreover, fucoxanthin inhibited the multiplicity of CRC in mice by inducing apoptosis, evidenced by a high number of cleaved caspase-3 cells in colonic adenocarcinoma and mucosal crypts [37]. Terasaki et al. suggest that fucoxanthin prevents CRC by inducing anoikis, apoptosis caused by the disruption of the anchorage to the extracellular matrix or adjacent cells, and by suppressing integrin β1, p-FAK, and p-Paxillin in mice colonic crypt cells [38].

Carotenoids can participate in potentiating the chemotherapy effect when it is combined with 5-FU. Fucoxanthin and phloroglucinol potentiate the cytotoxic effect of chemotherapy in colon cancer cells without harming normal cells [39]. Fucoxanthinol, a fucoxanthin derivative, impeded cell proliferation by downregulating pAkt, PPARβ/δ, and PPARγ in the colonospheres formed by human CRC cells [40]. Crocin, another carotenoid found in saffron, has antiproliferative, antiangiogenic, and antimetastatic properties in CRC via TNF-α, NF-kB, and VEGF pathway suppression in CRC cells [41]. It also increases glutathione synthesis, enhances superoxide dismutase, and eliminates ROS [42]. It is also considered a bioactive compound against the depression [43] present in a high percentage of oncologic patients that can contribute to the adherence and response of oncologic treatment.

Regarding controversial findings in the literature, CRC risk reduction in the population through a higher dietary intake of polyphenols and carotenoids may be negligible compared to other risk factors; nevertheless, these findings could become significantly beneficial to higher-risk individuals. For example, people with inflammatory bowel disease (IBD), such as ulcerative colitis, who are predisposed to CRC, may have a more significant cancer risk reduction from diet modification than people without IBD. Studies have shown the potential cancer prevention effects of polyphenol-rich strawberries and black raspberries in IBD-related CRC, perhaps an intervention with low toxicity that can supplement current IBD treatment [44]. 

Research in regulating the metabolic pathways that promote the proliferation of tumor cells through dietary compounds may find more consistent results by focusing their studies on higher-risk populations. Another potential area of research is the synergistic anticancer effects of polyphenols and carotenoids. They are usually studied independently; however, dietary intake commonly includes compounds from both families, meaning their therapeutic benefits could derive from the chemical interactions between polyphenol and carotenoid structures.

## 3. Regulation of Molecular Pathways in Colorectal Carcinogenesis by Bioactive Compounds

A distinct hallmark of cancer is metabolism pathways, which is why research is focused on knowing how to regulate the metabolism pathways related to CRC, potentially using this knowledge to improve current treatments. The Wnt pathway, best known as Wnt/B catenin, participates in cancer initiation and progression and regulates the pluripotency of cells during development and differentiation [45]. The invasion and prognosis of metastasis are related to the epithelial–mesenchymal transition (EMT), the malignant proliferation of tumor cells, decreased apoptosis, and the regulation of expression of EMT markers. A loss of E-cadherin expression is a molecular key point [46]. Another molecular mechanism for developing CRC is the signal transducer and activator of transcription (STAT). In this regard, seven STAT factors share molecular characteristics that control their action mode; specifically, STAT1 and STAT3 have an important role in CRC progression [47]. Moreover, cytokines such as IFN-γ, IL-6, IL-1, and EGF [48] activate a family of proteins associated with their receptors, the Janus kinases (JAK), which activate STAT molecules. The most studied in CRC is JAK2/STAT3 since they relate to EMT. The JAK/STAT pathway promotes invasion, migration, growth, and chemoresistance [49].

The tumor microenvironment is the different cell types surrounding the tumor, including fibroblasts, endothelial cells, macrophages, dendritic cells, tumor stem cells, adipocytes, and the microbiome. They are key in promoting or restricting tumor development [50,51,52]. The changes that occur in the tumor microenvironment resemble a state of chronic inflammation, which begins with ischemia that continues to interstitial edema, the infiltration of immune cells, and angiogenesis, suggesting that the tumors are infiltrated by inflammatory cells and cytokines PDGF, EGF, IL-1, TNFα, and TGFβ [53] that favor oxidative stress and the presence of ROS [54]. 

CRC begins with altering the regulatory mechanisms of DNA repair systems and the cell proliferation of mucosa cells lining the colon and rectum. These cells convert into neoplasia that develops polyps that advance to high-grade dysplasia and evolve into invasive tumors. Early genetic mutations include the BRAF and APC genes [55].

CRC develops due to cancer stem cells (CSC), pluripotent stem cells with self-renewal capacity to different lineages found in the colon, promoting carcinogenesis and favoring tumor heterogeneity [56]. Microbiota and metabolome alterations are involved in pro- or anti-cancer actions [57]. Several phytochemicals involved in regulating the Hedgehog, Notch, and Wnt/β-catenin pathways, such as curcumin, quercetin, lycopene, cinnamic acid, resveratrol, sibylline, and EGCG, have been identified. Their importance in regulating pathways as they regulate the maintenance and proliferation of CSCs is summarized below (Table 2). The inflammation process promotes cancer progression through inflammatory cytokines in the tumor microenvironment, such as TNF-α, which is a mediator involved in chronic inflammatory diseases with greater participation during the early stages of carcinogenesis, angiogenesis, invasion, generating reactive oxygen, and nitrogen species [58,59]. Also, cytokines such as IL-2 and IL-6 favor tumor proliferation, inhibit apoptosis, and participate in the conversion of non-cancerous cells to tumor stem cells. Moreover, TGF-b improves invasion and metastasis by inducing the transition epithelium-mesenchymal cells and IFN-γ participate in metastasis [60]. Lastly, Wnt signaling is involved with NF-ĸB and MAPK; together, they can increase oxidative stress and inhibit apoptosis [61,62]. 

Curcumin and quercetin are the most studied bioactive compounds regarding CRC. On the one hand, curcumin is a natural and active derivative of turmeric. It is an oily molecule soluble in acetic acid and ketones but appears insoluble in water. It possesses anti-angiogenic, anti-tumor, antiproliferative, and anti-inflammatory properties through various mechanisms, including the suppression of intrinsic and extrinsic apoptotic signaling pathways, cell cycle arrest, and the activation of autophagy. Curcumin, obtained from the dried root of Curcuma longa, can kill only tumor cells without harming healthy cells due to a high expression of a protein known as GADD45a (the gene activated during DNA damage). However, there are limitations in its use as a therapeutic agent since it has low solubility, poor absorption, rapid metabolism, and rapid elimination, which is why the use of nanoparticles has been implemented to encapsulate it so that it can achieve its therapeutic effect within the tumor [89,90] On the other hand, Quercetin is a secondary metabolite of plants. It has an anti-tumor effect by regulating the signal transduction pathways to prevent, inhibit or reverse carcinogenesis. It also has anti-inflammatory and antioxidant effects. It may potentiate cytotoxic effects or reduce the side effects of chemotherapeutic drugs on normal cells or reverse drug susceptibility [90]. 

Numerous health benefits of these food supplements are recognized by pioneering experimental studies involving both in vitro and in vivo studies in recent decades, including their antioxidant and anti-inflammatory potential, digestive stimulant effects, hypolipidemic actions, antilithogenic properties, anti-diabetic influence, antimutagenic, and anticancer potential [91]. Studies have shown that spices and their bioactive compounds can inhibit or even activate the pathways related to cell division, proliferation, and detoxification, in addition to having immunomodulatory and anti-inflammatory effects [92].

## 4. Chemosensitive and Chemopreventive Properties by Bioactive Compounds in CRC

There are several applications for polyphenols and carotenoids that have not been sufficiently explored, such as their potential use for predisposition to CRC [93,94], sensitizers to current CRC standard chemotherapy [23,39] candidates for treatment [95,96], and the reduction in its recurrence [97]. Using polyphenols and carotenoids as sensitizers and buffers to chemotherapy and radiotherapy could decrease drug resistance and minimize toxicity by requiring lower doses [98,99]. Further research into the adjuvant treatment of CRC with dietary polyphenols and carotenoids may yield improved outcomes for patients with lower costs and minimal risks.

**Figure 1:**
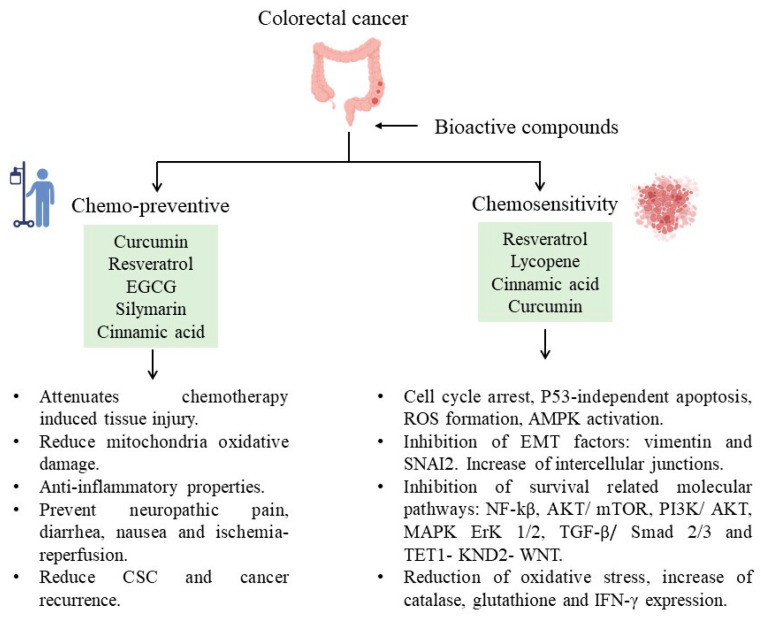
Bioactive compounds and their participation in decreasing CRC.

These bioactive compounds have recently been found to protect against chemotherapy side effects or modify susceptibility by reversing chemoresistance (Figure 1). For example, curcumin attenuates oxaliplatin-induced liver injury by activating nuclear erythroid factor 2-related factor 2 (Nrf2) signaling, regulating cellular defense pathways, and the oxidative damage to mitochondria caused by oxaliplatin [100]. Furthermore, curcumin protects against irinotecan-induced intestinal injury inhibiting NF-ĸB activation [101]. It is also active against FOLFIRI and bevacizumab cardiotoxicity by suppressing oxidative stress and preventing mitochondrial dysfunction in cardiac mitochondria [102]. Regarding quercetin, reports from specific cell lines (SW620) exposed to this bioactive compound appeared to be more sensitive to doxorubicin due to the inhibition of ATP-driven transport activity of P-glycoprotein that leads to a higher concentration of doxorubicin at the intracellular level. Also, it has been proposed that quercetin can reverse multidrug resistance by regulating the expression of glutamine transporter 1 member 5 (SLC1A5) [103]. 

The studies of resveratrol against cancer have demonstrated chemopreventive functions related to anti-inflammatory, antioxidant, anti-apoptosis, and anti-proliferative properties [104,105,106,107]. Resveratrol may reduce the side effects of chemotherapy, such as renal toxicity, cardiotoxicity, gastrointestinal toxicity, hepatotoxicity, and UVR-induced skin cancer [108]. In addition, resveratrol has been used as a chemosensitizer in CRC cells (HCT116, HT-29, and SW620) to 5-FU by inducing cell cycle arrest and apoptosis independently of p53 status and inhibiting their endogenous antioxidant capacity, respectively [109,110]. In a study with etoposide-resistant HT-29 cells, resveratrol was a chemosensitizer, inducing cell cycle inhibition, ROS formation, AMPK activation, and apoptosis induction [111]. The activation of AMPK and SIRT1 has long been thought to be the mechanism via which dietary bioactive compounds influence their health benefits. According to Liu et al., resveratrol inhibits proliferation and induces death in ovarian cancer cells (A2780 and SKOV3) by reducing glycolysis and targeting the AMPK/mTOR signaling pathway [112]. In breast cancer, resveratrol regulates EMT by modulating TGF-β1. Furthermore, resveratrol can induce autophagy by upregulating SIRT3 and phosphorylated AMPK, suggesting that the resveratrol-mediated inhibition of tumor progression is attributed to the participation of the SIRT3/AMPK/autophagy signaling axis [113]. Another example is curcumin-induced AMPK and its downstream factor ACC phosphorylation in lung cancer cells [114,115] and prostate cancer [116]. EGCG promotes cell survival by shifting the balance of the mTOR-AMPK pathway during ER stress [117]. The indirect AMPK activators show promise in treating various disorders such as cancer. The effects of systemic and chronic AMPK activation remain unknown.

Resveratrol also sensitizes CRC cells to 5-FU by inhibiting EMT factors (vimentin and SNAI2 proteins), increasing intercellular junctions (desmosomes, gap, and tight junctions, and adhesion molecules such as E-cadherin), and inhibiting the NF-kB pathway. Also, it has been reported that this compound downregulates p-AKT in cancer cell lines; however, it also upregulates the same pathway to prevent paclitaxel-induced neuropathic pain or ischemia-reperfusion injuries [118]. The combined treatment of 5-FU and resveratrol in colorectal cancer sensitizes tumor cells to 5-FU, which induces a further increase in oxidative stress related to the inhibition of the AKT and STAT3 proteins [119] known for their oncogenic potential in colorectal carcinomas [120].When resveratrol is combined with forskolin, it has phosphodiesterase 4D inhibitory effects to inhibit AKT/mTOR signaling in colorectal cancer cells [121]. In the same polyphenol family, EGCG can decrease the probability of recurrence of colorectal adenocarcinoma [122] in combination with cisplatin or oxaliplatin, evidenced by an improved therapeutic effect. 

Another chemosensitizer mechanism to 5-FU by resveratrol is through miR-34 regulation. This mechanism was studied in vitro using HCT116 cell lines. The mechanism was mediated by inhibiting the PI3K/Akt and MAPK Erk1/2 signaling pathways that increased miR-34a production and thus indirectly suppressed the *SIRT1* gene via *E2F3* gene expression [123,124]. Also, a study found that resveratrol sensitizes HT-29 and HCT-116 CRC cells to oxaliplatin, increasing the expression of miR-34c, which inhibits its target, KITLG [125]. Moreover, when resveratrol and oxaliplatin are combined, the cell proliferation of Caco-2 CRC cells decreases due to the induction of apoptosis and necrosis [126].

Previous studies have reported that consuming lycopene during cancer treatment may reduce the oxidative stress caused by 5-FU and oxaliplatin treatments, reducing the associated side effects. It has also been proposed as a chemosensitizer due to the possibility of inducing the expression of apoptotic genes [35]. In addition, lycopene enhances parameters such as catalase, glutathione, and IFN-γ expression in the presence of 5-FU, which may activate antitumor effects and further enhance the effect of 5-FU on cancer elimination [34]. In this regard, in 2021, it was reported that silymarin supplementation could reduce the occurrence of side effects such as diarrhea and nausea in patients with CRC receiving FOLFIRI as first-line treatment plus bevacizumab; however, it does not show a hepatoprotective effect [127]. 

According to studies, cinnamic acid has cardioprotective [128] and hepatoprotective activity and an analgesic effect during chemotherapy [129]. For example, an in vivo model with rats (Sprague Dawley) treated with oxaliplatin developed side effects; meanwhile, when cinnamic acid was provided, neuropathic pain decreased [130]. 

There are a few studies of cinnamic acid and CRC as a chemosensitizer; however, it has been recently demonstrated that cinnamic acid alone or combined with FOLFOX reduces side population cells and cancer stem cell markers in the HT29 cell line [73]. However, the chemosensitizer potential of cinnamic acid in chemotherapy has not been explored, so we consider it a promising candidate for study.

Curcumin can reverse chemoresistance by upregulating EMT markers by attenuating the TGF-β/Smad2/3 signaling pathway or downregulating TET1-NKD2-WNT [131]. Curcumin as an adjuvant treatment to FOLFOX chemotherapy in CRC patients did not decrease neurotoxicity or quality of life but improved overall survival in the curcumin group versus those with only chemotherapy [132]. However, in vitro curcumin has been able to sensitize colon cancer stem cells to chemotherapy drugs such as 5-FU, FOLFOX, and irinotecan, further reducing the emergence of chemoresistant cells [70]. In addition, curcumin and resveratrol have been linked to the ability to prevent breast metastasis and gastric cancer, osteosarcoma, and lung cancer through the coordinated action of several molecular pathways such as the SIRT3/AMPK/autophagy signal axis, JAK2/STAT3, and MALAT1/miR-383-5p/DDIT4 [113]. Quercetin involves reduced metastasis by negatively regulating the expression of the protein survivin, which is expressed in the macrophages that regulate the processes of proliferation and cell death. This bioactive compound also regulates cyclin D1, which controls the cell cycle.

## 5. Conclusions

CRC has a high incidence and is the second most deadly cancer worldwide. However, it is largely preventable and treatable, and nutrition plays a pivotal role. Dietary bioactive compounds in foods such as vegetables, fruits, seeds, nuts, oils, and tea are important in cancer prevention since they may regulate cell differentiation processes, proliferation, and apoptosis; all are affected in cancer cells. Bioactive compounds regulate these molecular pathways related to inflammatory diseases like CRC. 

Polyphenols and carotenoids are the most studied bioactive compounds due to their antioxidant, anti-inflammatory properties, and their modulation of gut microbiota. Also, a higher intake of total bioactive compounds, such as phenolic acid, anthocyanin, and flavonoids, has been related to a reduced CRC risk, acting as preventive compounds. The positive evidence about bioactive compounds promotes their study as a strong line of research, and the knowledge obtained could improve the quality of life of patients and reduce all the associated effects of chemotherapy, such as renal, cardio, gastro, and hepatotoxicity, making these compounds an important part of the prevention and treatment of CRC.

The diverse molecular pathways related to CRC, like JAK/STAT, Wnt/β catenin, EMT, PI3K/AKT, and mTOR, can be regulated by bioactive compounds promoting apoptosis, inhibiting or decreasing cell growth and proliferation, reducing inflammation, eliminating cancer stem cells, and avoiding chemoresistance. Even though all the bioactive compounds have different mechanisms of action, many share mechanisms, such as the regulation of pro- or anti-apoptotic proteins, the modulation of the tumor microenvironment, the regulation of the expression of efflux pumps, and a reduction in oxidative stress.

Since these compounds have an antitumoral effect, their potential has been explored with oncologic treatments, including monoclonal antibodies and CRC first-line chemotherapy, 5-FU.

We consider that good nutrition, represented by consuming bioactive compounds, can benefit oncologic patients. Nevertheless, there are limitations to consider, such as the metabolite status, the administration form, and the physiological status of the patients. These may influence the correct biotransformation, utilization, and beneficial effects. Another limitation is the low number of studies demonstrating the chemosensitivity of these bioactive compounds. Several investigations have only reported in vitro assays with cancer cell lines. There are still important questions about bioactive compounds and their action in cancer, which is why carrying out research with primary culture cells is relevant in order to evaluate the potential of bioactive compounds at different doses and determine their effectiveness.

## Figures and Tables

**Table 1:** Bioactive compounds and food sources.

Natural Products	Bioactive Compound	Food Sources
Flavonoids	Curcumin	Turmeric, Ginger, Curry
	Resveratrol	Red wine, Red grapes, Peanuts
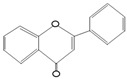	Quercetin	Onions, Tea, Apples, Kale
	Epigallocatechin Gallate	Green tea, White tea, Black tea
	Anthocyanins	Blackberries, Raspberries, Cherries
Phenolic Acids	Caffeic Acid	Coffee beans, olives, potatoes, carrots, propolis
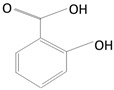	Ellagic Acid	Pomegranates, blackberries, raspberries, strawberries
	Gallic Acid	Grapes, strawberries, blueberries, mango, plums, hazelnut
Carotenoids	α-carotenoid	Carrots, sweet potatoes, pumpkin, broccoli, spinach
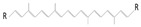	β-carotenoid	Carrots, sweet potatoes, pumpkin, spinach, kale
	Lycopene	Tomatoes, watermelon, grapefruits
Xanthophylls	β-cryptoxanthin	Citrus fruits, papaya, egg yolk, apples
	Astaxanthin	Seafood, tomato
	Fucoxanthin	Brown seaweeds
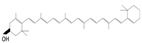	Lutein	Broccoli, spinach, kale, kiwi, grapes, pumpkin
	Zeaxanthin	Broccoli, spinach, kale, orange, peppers

**Table 2:** Bioactive compounds and their action in metabolic pathways in CRC.

Metabolic Pathway	Bioactive Compound	Mechanism	Function	Reference
↓PI3K/AKT/mTOR	Curcumin	↓miR 21/PTEN/Aktand IL-8 ↓AP-1 Activity	Inhibits growth, cell proliferation, and migration. Promotes apoptosis.	[63,64]
	Quercetin	↓p-AKT and Bcl-2 levels ↓pGSK3β	Decreases cell growth, proliferation, and migration.	[65,66]
	Resveratrol	↓PI3K/PTEN ↓ AKT ↓ IL-8	Decreases colon cell proliferation and formation. Regulates induced cell apoptosis (chemoprotection) and arrest in the G1 phase.	[67,68]
	Lycopene	↓p-AKT ↓Cyclin D1expression	Inhibits cell proliferation.	[69]
↓EMT	Curcumin	↑E-cadherin ↓Twist, vimentin	Reduces chemoresistance. Expression of BMI1, SUZ12, and EZH2 transcripts Suppresses migration and invasion.	[70]
	Quercetin			[71]
	Lycopene	↑E-cadherin Inhibits PCN, and AP-1	Reduces chemoresistance	[72]
	Cinnamic Acid	In combination with FOLFOX: ↓OCT4 ↓NANOG ↓ABCB1, ↓ALDH1A1	Eliminate cancer stem cells.	[73]
↓Wnt/β-catenin	Curcumin	Acts as a PPARγ receptor agonist.	↓ Inflammation TNF-α, growth, PGE2 levels	[71,74]
	Quercetin	↓MMP-2 ↓MMP-9 ↓TLR4 ↓NFKB ↓TNFα ↓COX-2 ↓IL-6		
	Resveratrol	↑IL-1β ↓Str1 ↓TGFb ↓NFKB ↓SUMO ↓WNT	Decreases invasion and inflammation.	[75]
			Reduces proliferation, formation of colonies, and invasion. Decreases spheroid formation and expression of CSC markers.	
	EGCG	Promotes proteasome phosphorylation and degradation of β-catenin through a GSK-3B and PP2A-independent mechanism.	Decreases cell proliferation and induces apoptosis. Inhibits autophagy. Reduces inflammation.	[76,77,78]
	Lycopene	↓CoX-2 ↓PGE2 ↓IL-1β ↓IL-6 ↓TNF-α ↓iNOS	Reduces invasion.	[79]
		↓β-catenin ↓c-Myc ↓MMP-7 ↓MMP-9	Regulate cell cycle, survival, autophagy, angiogenesis, and inflammation.	
	Silibinin	↓COX-2 ↓NFKB ↓IL-6 ↓TNF-α ↓D1cyclin ↓Bcl-2 ↓VEGF ↓MMP2 ↓iNOS	Cell cycle regulation and apoptosis	[80]
	Quercetin	Inhibits D1 Cyclin, survuvin and GSK3		[81,82]
	Resveratrol	↑Superoxide dismutase ↑glutathione peroxidase Sirt1/AMPK activation		[83]
Apoptosis	Curcumin	↑ROS ↑Bax ↑caspases	Promote apoptosis	[84]
	Lycopene	↑DR5, Fas ↑p21 ↑Bax1, ↑caspase 3		[85]
	Cinnamic Acid	↑Phase G0-G1 cells ↓PhaseS, G2/M cells ↑ROS ↑p21 ↑caspase 3	Promote apoptosis	[86]
JAK/STAT	Quercetin	Cell arrest in G0/G1 by p-STAT3	Promote apoptosis and necrosis.	[87]
		↑Bax, ↑caspase3 ↑p53		
	EGCG	↓Ki67 ↓promoter activity and transcription of STAT3 Inhibits AKT, ERK1/2 o P38 MAPK ↑Caspases-3, PARP, Bcl-2, Bim, Bak, MCL-1, E-cadherin, and Vimentin	Reduces proliferation and migration. Inhibits cell proliferation and promotes apoptosis.	[88]

Arrow meaning: ↑ Increase, ↓ Decrease.

## Data Availability

Not applicable.

## Abbreviations

5-FU	5-Fluorouracil
ABCB1	ATP binding cassette gene B1
AKT	Serine/threonine kinase
ALDH1A1	Aldehyde dehydrogenase 1A1
AP-1	Activator protein-1
APC	Adenomatous polyposis coli
BAX	Bcl-2 Associated X-protein
BCL-2	B-cell lymphoma 2
Bim	Bcl-2-interacting mediator
BRAF	V-Raf Murine Sarcoma Viral Oncogene Homolog B oncogene
COX 1	Cyclooxygenase 1
COX 2	Cyclooxygenase 2
CRC	Colorectal Cancer
CSC	Cancer Stem Cells
DNA	Deoxiribonucleic acid
DNMT3A	DNA methyltransferase 3 alpha
DR5	Death Receptor 5
EGCG	Epigallocatechin-3-gallate
EMT	Epithelial Mesenchymal Transition
ERK	Extracellular Signal-Regulated Kinase
FOLFIRI	Folinic acid, Fluorouracil and Irinotecan
FOLFOX	Folinic acid, Fluorouracil and Oxaliplatin
GSK-3	Glycogen Synthase Kinase-3
IBD	Inflammatory Bowel Disease
IFN-γ	Interferon gamma
iNOS	inducible Nitric Oxide Synthase
JAK	Janus Kinases
KITLG	c-KIT ligand gene
MAPK	Mitogen-Activated Protein Kinases
MCL-1	Myeloid Leukemia 1
MiRNA	Micro RNA
MMP-2	Matrix Metalloproteinases 2
MMP-9	Matrix Metalloproteinases 9
mRNA	Messenger RNA
mTORC	mechanistic Target Of Rapamycin Complex 1
NF-kB	Nuclear Factor Kappa-light-chain-enhancer of Activated B cells
Nrf2	Nuclear Erytroid Factor 2 related factor 2
OCT 4	Octamer-Binding Transcription Factor 4
PARP	The Poly (ADP-ribose) Polymerase
PCN	Phenazine-1-carboxamide
p-FAK	Phospho- Focal Adhesion Kinase
PGC1α	Peroxisome Proliferator-activated receptor-gamma Coactivator α
PGE2	Prostaglandin E2
PI3K	Phosphatidylinositol 3-kinase
PPARβ	Perosyxomel Proliferator Activated Receptor β
PPARδ	Perosyxomel Proliferator Activated Receptor δ
PPARγ	Perosyxomel Proliferator Activated Receptor γ
PTEN	Phosphatase and Tensin homolog
ROS	Reactive Oxigen Species
SIRT1	Silent Information Regulator 1
SLC1A5	Solute Carrier Family 1 Member 5
STAT	Signal Transducer and Activator of Transcription
STR1	Stromelysin-1
SUMO	Small Ubiquitin-like Modifier
TGF-β	Transforming Growth Factor beta
TLR4	Toll Type Receptor 4
TNF-α	Tumor Necrosis Factor Alpha α
TSC1	Tuberous Sclerosis Complex 1
TSC2	Tuberous Dclerosis Complex 2
UVR	Ultraviolet Radiation
VEGF	Vascular Endothelial Growth Factor
WNT	Wingless-related integration site
